# An Account of a Chemico-Gelatinous Injection, with the Method of Employing It in Anatomical Preparations

**Published:** 1850-03

**Authors:** Henry Goadby

**Affiliations:** Late Dissector of Minute Anatomy to the Royal College of Surgeons of England


### RECORD OF MEDICAL SCIENCE.

### ANATOMY AND PHYSIOLOGY.

*An Account of a Chemico-Gelatinous Injection, with the method of employing it in Anatomical Preparations.* By Henry Goadby, Esq., F. L. S., late dissector of Minute Anatomy, to the Royal College of Surgeons, of England.

At the latter part of the year 1841, I was desirous of making a series of minutely-injected preparations to illustrate the peculiar arrangement of the capillary vessels in the different tissues of the human subject. My experience of the materials usually employed for this purpose—i. e., common size, or parchment size, colored with vermillion, left but one impression on my mind—viz., that the particles of the finest vermillion are too coarse to pass through the capillary system. Occasional *patches* of tissue are met with, *beautifully injected*, while all around is a mass of extravasation, and this remark applies especially to *fœtal* subjects.

Prior to the above date, M. Gruby published an account in the *Comptes Rendus* of some very successful injections which he had made by employing certain fluids, which he used separately, and which, when they met, mutually decomposed each other, and deposited the coloring matter in the vessels themselves.

He used saturated solutions of the chromate or bichromate of potash, and of the acetate of lead: he directed that equal quantities of these fluids should be used, first injecting all the chromate of potash, to the extent of one half the quantity of injection supposed to be necessary, into the vessels, and subsequently the same quantity of the acetate of lead.

As soon as these fluids meet, they decompose each other; the acetic acid of the acetate of lead combining with the potash to form the acetate of potash, which is set free, and the chromic acid of the chromate of potash combining with the lead to form the beautiful chromate of lead, which is deposited in the vessels.

The reports which had reached me were highly confirmatory of M. Gruby's success with these fluids, and having seen Mr. Bowman's preparations of the kidney injected on this principle, and with the like materials, I determined to employ them. My experiments, however, were most unsatisfactory, for, having injected a terrier puppy, a dissection of several hours was required to ascertain whether I had succeeded in injecting any part or not, and my best reward consisted in a patch of capillaries, slightly painted of a pale yellow color, and entirely wanting that roundness and fulness, characteristic of a good injection.

I next procured a human fœtus, and injected it, with precisely the same results.

On making inquiry of Mr. Bowman, touching the ordinary success which had attended his experiments, and the experiments of others, so far as he knew, he told me that I appeared to have met with fair average results, for that the labor of dissecting, consequent on using these fluids, was always great, and that the operator must consider himself well rewarded for two or three days' work, by finding a microscopic bit well injected.

From this narration of failures, it will be evident that the fluids *rarely meet* in the vessels, otherwise the color would be necessarily precipitated. With a view to see exactly what took place, I determined to inject a piece of intestine, in which the whole process would be under my inspection. I placed pipes in the mesenteric veins, and secured all the cut vessels in the usual manner; I then proceeded to throw in the chromate of potash, and found that the potash would not wait for the lead, but came out instantly through the parietes of the vessels as fast as it went in, and in one broad stream covered the table. I repeated this experiment a number of times, but with the same uniform result; on some occasions I threw in the lead also, and as the vessels were moist with the chromate, the slight painting I have mentioned took place; but as only equal quantities of the two fluids produce the best color, the excess of the lead was useless.

Having observed, at this stage of my experiments, that the precipitated chromate of lead is remarkably fine and soft, I determined to use it, in lieu of vermillion, with size; and although the success was far greater than when I used the fluids separately, the results were in no way superior to the old red injection.

The principle involved in M. Gruby's use of these fluids: that, namely, of forming the color within the vessels themselves, appeared to be undeniably good, notwithstanding it had so signally failed in my hands, and, as far as I could learn, in the hands of all those persons who had hitherto employed it; and I had no doubt that if I could succeed in giving some consistence to the fluids, the results might prove more satisfactory. For this purpose, size would not do, as it is rarely, when bought, much too strong for use, and it would not bear further dilution. I therefore procured the highly concentrated preparation employed by pastry cooks, and sold by grocers, under the name of gelatine. The following is my formula for the double injection with this material:—

Sat. solution of bi-chromate of potash, eight fluid ounces; water, eight ounces: gelatine, two ounces.

Sat. solution of acetate of lead, eight fluid ounces; water, eight ounces; gelatine, two ounces.

Thus, gelatine, two ounces, are dissolved in sixteen ounces of fluid, and kept and used separately as before; but the success consequent on the addition of the gelatine was quite extraordinary; the vessels were all full and round, and there was no extravasation; and for reasons hereafter to be explained, the microscope revealed scenes so rich in depth, color, and beauty, as to exceed the best red injections I have ever seen.

With this form of injection I have never failed; I have injected three fœtal subjects so minutely, that the capillaries of the skin, and of every tissue, were perfectly injected. Amongst the best specimens I obtained, I may mention injections of the papillæ of the lips, gums, and tongue; of the pulps and capsules of the teeth; of the conjunctivæ and other tissues of the eye; of the mucous membrane of the nose and cellular tissue; fascia; periosteum, &c.; ceruminous glands, lymphatic glands, and thyroid glands; pericardium, auricles of the heart, vasa vasorum, particularly of the aorta and vena cava, and the vessels of all the nerve sheathes. In fine, one fœtus occupied me in dissecting, ten hours a day, for two months, and was scarcely half finished at the expiration of the time.

Having described the success attending the use of these injecting fluids, I must now say how they are to be mixed and used, as everything depends on care in these respects.

Each parcel of gelatine must be dissolved in the water only, (eight ounces,) and in a separate water-bath. The water-baths I employ, consist of two earthen pans, such as are applied to a child's chair, and capable of containing about one quart each; these are fitted to two tin kettles, the broad flange of the earthen pan resting on the rim of the kettle, the pan covered with a common saucepan lid. The kettles should be furnished with a bail of iron wire, like that of a glue-pot, or pitch-kettle.

The gelatine is to be slowly dissolved in the eight ounces of water; when this is accomplished, the eight fluido unces of bi-chromate are to be added to the gelatine in one pan, and the eight fluid ounces of acetate of lead to the gelatine in the other; each should be well mixed by stirring with a glass rod, a separate rod being used for each solution, lest the chromate of lead should be precipitated.

The fluids thus prepared, must then be strained through fine flannel (using a piece for each fluid) into other vessels, the earthen pans cleaned, and the fluids returned to them. The injections are now ready for use, and must be kept at a temperature of about 90° by the warm water contained in the kettles.

*Directions for Using the Injection.*—The best subject to inject is a fœtus, as there are no cut vessels by which the injection can escape. A pipe, with a stop-cock attached, should be firmly tied in the umbilical vein, leaving the arteries open until the yellow injection makes its appearance, when they should be secured. It is most essential that, for this injection, the subject be warmed through by immersion in warm water, the temperature of which must not be higher than 90°, or corrugation of the tissues will take place; it will require from one hour to two hours to accomplish this, and the temperature must be maintained until the injection be completed. The whole sixteen ounces of the potash preparation of gelatine must now be used, care being taken that its temperature never exceed 90°. Some manipulators deem care of little import in the early stage of injecting, and throw in the first few syringefuls rapidly, and only exhibit caution when the subject begins to fill. In my experience, this is an error; and he who would succeed must be equally careful and patient throughout. It is my practice to let the piston descend so slowly, that it can scarcely be seen to move.

Having used the whole of the first preparation, the acetate of lead must be used, when the color will instantly be formed, and give the operator some idea of his progress.

The temperature of the subject must be kept up, and a fresh batch of injection made and strained as before. In about half an hour the injection may be resumed, and the bi-chromate again claims precedence; but only half the quantity need be used now, followed by an equal quantity of the lead. At this point the stop-cock should be turned, and the subject again allowed to rest for half an hour; the remainder of the injections may then be used, and after this, in all probability, the subject will require another batch. The manipulator who employs for the first time, as much injection for a fœtus as I have already directed to be used, and who experiences the great resistance opposed to the transmission of the last several syringefuls, especially as the body will by this time be swollen and tense to an amazing degree, will feel somewhat surprised to learn, that if he suspend the operation for an hour, keeping up the temperature in the meanwhile, he will be able to throw into the subject twenty or thirty ounces more with comparative ease, and have the pleasure of seeing many isolated congeries of vessels of the skin gradually approaching each other, and finally anastomosing most perfectly, while the tension of the body will be so great, that if the piston be pressed completely down, and the hand withdrawn, it will gradually rise, and the same may, with care, be repeated several times, without causing extravasation.

Towards the conclusion of the process, the injections should be thrown in alternately; and this should be continued, notwithstanding the prodigious distortion of the body, as long as the injection is felt to flow in the vessels. To inject a fœtus well, on this plan, will occupy from four to five hours. The operation finished, the body should be thrown into cold water, and should not be dissected until the next day.

*The Dissection,*—Will soon reveal what has become of the injection, and is altogether a disagreeable and difficult task. It will be found that nearly all the gelatine and acetate of potash have transuded and separated the tissues widely from each other, and that the blood has been diluted, and intimately mixed with the gelatine, which is colored by it.

The majority of preparations thus injected, require to be dried, and mounted in Canada balsam. Each preparation, when placed on a slip of glass, will necessarily possess more or less of the colored infiltrated gelatine, which, when dry, forms, together with the different shades of the chromate of lead, beautiful objects, possessing depth and richness of color. The gelatine also separates and defines the different layers of vessels. By this injection, the arteries are always readily distinguishable, by the purity and brightness of the chromate of lead within them, while the veins are detected by the altered color imparted by the blood.

Those preparations which require to be kept wet, can be preserved perfectly in my B fluid, specific gravity 1,100; the A fluid destroys them. I regret much that a gentleman, (now deceased,) Alexander Nasmyth, Esq., for whom I prepared a great number of such injections, had no faith in my preserving fluids at that time, and employed a chemist to prepare a fluid for the preservation of these yellow injections, which has quite destroyed some of the most beautiful preparations I have ever seen, while some of the fellow parts in my possession, are as perfect now as they were seven years ago.

I would recommend, that the slips of glass employed for the dry preparations be instantly inscribed with the name of the preparation, written with a diamond, for, when dry, it is very difficult to recognise one preparation from another, until the operator's eye be educated to the effects of this chemico-gelatinous injection. Where so much wet abounds, gummed paper is apt to come off.

When dry, it is sufficient for the purpose of brief examination by the microscope, to wet the surface of a preparation with clean oil of turpentine; immediately after examination, it should be put away carefully in a box, to keep it from the dust, until it can be mounted in Canada balsam.

The bi-chromate of potash is greatly superior in color to the chromate, which yields too pale a yellow; and subsequent experience has convinced me that the acetate of potash frequently effects its liberation by destruction of the capillaries, and this, even long after the preparations have been mounted in Canada balsam; perhaps this may be owing to some chemical action of the acetate of potash upon them.

I would suggest the substitution of the *nitrate* for the *acetate* of lead, as we should then have, in the liberated nitrate of potash, a valuable auxiliary in the process of preservation.

Although highly desirable, as the demonstrator of the capillaries of *normal* tissues, I do not think this kind of injection fitted for morbid preparations, the infiltrated gelatine producing appearances of a puzzling kind, and calculated to mislead the pathologist.

In preparing portions of dried, well-injected skin for examination by the microscope, I have tried the effect of dilute nitric acid, as a corroder, with very good results. But probably, liquor potassæ would have answered this purpose better.

When size injection is to be employed, colored either with vermillion or the chromate of lead, the animal should be previously prepared by bleeding, to empty the vessels; for if they be filled with coagulated blood, it is quite impossible to transmit even size, to say nothing of the coloring matter. Hence the difficulty of procuring good injections of the human subject.

But with the "chemico gelatinous" injection no such preparation is necessary; and success should always be certain, for the potash liquefies the blood, while constant and long-continued pressure by the syringe drives it through the parietes of the vessels into the cellular tissue. The large quantity of infiltrated blood—the invariable concomitant of my process—characterizes this from all other modes of injecting, and is a distinctive feature of these preparations.

I find that a very superior preparation of gelatine is now on sale at at the grocers' shops, nearly equal in appearance to isinglass. From its apparent purity, I am led to infer that a less quantity of it would suffice for the number of fluid ounces mentioned above.

The only preparations of gelatine extant when I made the experiments recorded in my paper, contained a large quantity of dirty, insoluble gluten, from which defect I venture to assume the French gelatine is free.—*London Lancet.*

